# Shedding light on social dominance within the affective neuroscience personality scales

**DOI:** 10.1017/pen.2026.10007

**Published:** 2026-06-02

**Authors:** Kenneth L. Davis, Christian Montag

**Affiliations:** 1 Pegasus International, USA; 2 Centre of Cognitive and Brain Sciences, Institute of Collaborative Innovation, University of Macau, Macau SAR, China; 3 Department of Psychology, Faculty of Social Sciences, University of Macau, Macau SAR, China; 4 Department of Computer and Information Science, Faculty of Science and Technology, University of Macau, Macau SAR, China

**Keywords:** Panksepp, personality, social dominance

## Abstract

In the present paper we present psychometric properties on a Social Dominance scale from items which are embedded in the Affective Neuroscience Personality Scales 3.1. In two samples we show that psychometrics of the Social Dominance scale are sufficient and that correlations with scales measuring Panksepp’s primary emotional systems fail to consistently reach statistical significance with the exception of associations with ANGER (moderate effect size), SEEKING and CARE (small effect sizes). No associations with satisfaction with life and only small negative associations with Spirituality could be observed. We concur with Panksepp that Social Dominance is not a primary emotion but is a learned secondary emotion involving several primary emotions. We show evidence that Social Dominance is not the same as ANGER. We also briefly review mammalian (including humans) evidence that supports the view that Social Dominance is learned and also that the ANGER, SEEKING, and CARE primary mammalian emotions contribute to the learning of Social Dominance.

## 1. Introduction

Panksepp’s Affective Neuroscience Theory (ANT) sheds light on the emotional nature of mammals (Panksepp, 1998). By means of electrical brain stimulation, lesion studies and pharmacological challenge tests, he carved out seven primary emotional systems, which have been homologously conserved across the mammalian brain, because these systems are evolved tools for survival (Davis & Montag, 2019). The SEEKING, LUST, CARE and PLAY circuits represent the positive emotions mammals (including humans) experience, when the underlying neural circuits are stimulated. ANGER, FEAR and SADNESS represent the negative emotions, which are experienced when the respective neural networks are activated (Montag & Panksepp, 2017).

Due to ethical restraints, it is difficult to study the neural underpinnings of primary emotional systems in humans with techniques such electrical stimulation of the brain (although in general much progress has been made in the human neurosciences, for instance in the field of magnetic resonance imaging). In order to bring Panksepp’s AN theory to the tool box of psychologists and psychiatrists working with self-report measures, the Affective Neuroscience Personality Scales have been put forward aiming to assess individual differences as trait levels of the aforementioned primary emotional systems except for LUST (Davis et al., 2003).

Included in the latest version of the ANPS 3.1 are eight test items aiming to assess Social Dominance as a trait (Montag et al., 2021). In two recently published data sets, we investigated how Panksepp’s primary emotional systems measured with the latest version of the ANPS were linked to well-being as assessed with Diener’s Satisfaction with Life scale. In the present paper, we reused this openly available data set to shed light on the psychometrics of the eight items assessing Social Dominance and to relate them to Panksepp’s original AN theory.

There is a relatively long history of assessing trait dominance as part of a broader personality inventory. Raymond Cattell (1947) was the first to construct a dominance scale using factor analysis. In the 1947 paper, he published a dominance scale (Factor E, also included in his 16PF) based on dominant-assertive behaviors. Others followed: The Guilford–Zimmerman Temperament Survey (Guilford & Zimmerman, 1949) consisted of 10 scales the third of which was labeled “Ascendancy” and measured assertiveness and leadership qualities in social situations. Harrison Gough (1957) in his 20-scale California Psychological Inventory, included a Dominance scale associated with being dominant and forceful and Capacity for Status targeting being ambitious and wanting to be a success. The FIRO-B (Schutz, 1958) includes a nine-item scale labeled “Expressed Control,” which had items such as “I try to take charge of things when I am with people,” and “I try to have other people do things I want done.” The ANPS 3.1 Social Dominance scale included the assertiveness/taking charge, status seeking relative to peers themes as well as competitive/needing to win items.

The “Big-Five personality structure” grabbed the attention of the personality world as a robust, objective personality model (Goldberg, 1990). Panksepp’s primary emotions as measured by the ANPS correlated nicely with the Big-Five (Davis et al., 2003; Marengo et al., 2021), but the Big-Five did not have a Social Dominance scale. However, the Big-Five was constructed from adjective ratings, and Hofstee et al. (1992) published an analysis of where 540 adjectives loaded on Big-Five circumplexes. Goldberg ordered the Big-Five scales based on the amount of variance each scale accounted for in his factor analysis, and Extraversion was first scale and Agreeableness was second. Note, in our research, Big-Five Extraversion consistently aligned with PLAY, and Agreeableness aligned with CARE (Marengo et al., 2021). Importantly, Hofstee et al. (1992) showed that on the circumplex combining Extraversion and Agreeableness, adjectives like dominant, domineering, forceful, opinionated, and combative fell on the area of the circumplex circle defined by high Extraversion and low Agreeableness and adjectives like timid, unaggressive, and submissive fell on the opposite side of the circumplex, namely, low Extraversion and high Agreeableness. Our Social Dominance findings generally line up with this analysis, especially around the relationship between low Agreeableness and the CARE system.

In factor analyses, it is typical for dominance to load with Extraversion. For example, Harrison Gough (1987) reported a factor analysis of the 20 CPI scales on a sample of 1000 subjects, and the four highest loading scales on his Extraversion factor were Dominance (assertive, dominant), Capacity for Status (ambitious, wants to be a success), Sociability (sociable, likes to be with people), and Social Presence (self-assured, good talker).

In addition, the frequent similarity of play fighting and dominance fighting has led many to conceptually link social play with dominance formation. Panksepp suggested that while experience with the rough-and-tumble PLAY system is likely involved in the emergence of dominance, other primary emotions such as SEEKING, RAGE, and FEAR likely contribute as well (Panksepp & Biven, 2012, p. 169).

The scientific community discusses if Panksepp’s six primary emotions depicted here are exhaustive or if further brain systems anchored in subcortical brain regions are required to describe the emotional nature of mammals (note LUST is not discussed here). In this realm, it is discussed if (social) dominance should be seen as an additional distinct mammalian primary emotional system (also see van der Westhuizen & Solms, 2015).

In this paper, we offer the hypothesis that Social Dominance derives from three primary emotions: mainly (high) ANGER but also (high) SEEKING, and (low) CARE. However, we also provide evidence supporting the view that Social Dominance is not the same as ANGER and propose, similar to Panksepp, that Social Dominance is not a primary emotion but emerges from learning incorporating the ANGER, SEEKING, and CARE primary emotions. To support this view, we later discuss how the expression of Social Dominance has emerged phylogenetically in mammals including humans and likely hand-in-hand with the remarkable progressive expansion of the neocortex (Dunbar & Shultz, 2007), especially in primate evolution, which dramatically peaked in humanoids between 0.5 to 1.5 million years ago, long after humans split away from chimpanzees (Florio et al., 2016). In the discussion of the present findings, we also call attention to commonalities in how Social Dominance is expressed in chimpanzees and humans and highlight socially coercive and “bullying” versus prosocial cooperative styles of expressed Social Dominance.

## 2. Methods

### 2.1. Participants

Two international samples were recruited via the project website www.anps-research.com as in detail described in the recent paper by Davis and Montag (2024) – data are openly available. The first (final) sample consisted of 425 participants stating to be native English speakers (237 females; 188 males; mean-age: 39.80, SD = 16.04). A second (final) sample consisted of fluent, but non-native English speakers consisting of *N* = 338 (206 females, 132 males; mean-age = 32.93, SD = 12.38). More information on the background of the sample and the data cleaning processes can be found in the work by Davis and Montag (2024).

### 2.2. Affective neuroscience personality scales 3.1

All participants filled in the recent version of the ANPS consisting of 112 items. Aside from the assessment of six of the seven primary emotional systems (without LUST), each ANPS 3.1 scale has 14 items; one scale assesses spirituality with 12 items. Spirituality does not represent a primary emotional system but was added due to therapeutic reasons (to accommodate work with Alcoholics Anonymous). Embedded in the ANPS 3.1, eight items can be found assessing the scale we call “Social Dominance.” Note that four items need to be reversed before the scale can be summed (or mean-ed).

The items of the Social Dominance scale are depicted in Table 1. Internal consistencies for the Social Dominance scale were as follows: in the native-sample 1, *α* = 0.75; in the non-native, but fluent English sample 2, *α* = 0.75, both indicating acceptable consistency in sampling acknowledged elements of Social Dominance (see discussion above). Please note that internal consistencies are better without item 32 (sample 1: 0.78; sample 2: 0.77), hence future research might want to consider to not use this item further. We therefore also report the key correlations between Social Dominance and primary emotional systems in the supplementary material (no large difference). Supporting the Social Dominance scale’s construct validity, in a proprietary sample of job applicants, the ANPS Social Dominance correlated 0.473 with the FIRO-B “Expressed Control” scale (*n* = 870).

**Table 1.** Items of the social dominance scale being embedded in the ANPS 3.1

Item 08 (+)	I like to be the one in a group making the decisions.
Item 40 (+)	When I play games, it is important for me to win.
Item 88 (+)	I am not satisfied unless I can stay ahead of my peers.
Item 103 (+)	When working on a project, I like having authority over others.
Item 32 (−)	I prefer to enjoy participating rather than take the lead in group work.
Item 56 (−)	If my peers have outperformed me, I would still be happy, if I have nearly met my goals.
Item 72 (−)	When I play games, I do not mind losing.
Item 111 (−)	Striving to be better than my peers is not important for me.

Item 32 was modified from “I prefer to watch and observe than take the lead in group work” (van der Westhuizen & Solms, 2015) to the wording “I prefer to enjoy participating rather than take the lead in the group”.

An item correlation matrix for sample 1 is shown in Table 2. The items are arranged to highlight three highly correlating pairs of items in sample 1 representing Social Dominance themes: “liking to be in charge” (items 08 and 103), “needing to win games” (items 40 and 72), and “need to outperform peers” (items 88 and 111). These three highly correlating patterns were replicated in sample 2 as shown in Table 3. The correlation matrix for each sample shows that these three themes are interrelated and not statistically independent of each other.

**Table 2.** Social dominance item correlations, sample 1, with the correlations of 3 highly correlated pairs of items in bold

		008	040	088	103	032	056	072	111
ANPS_008	Pearson’s *r*	–	–	–	–	–	–	–	–
	*p*-Value	–	–	–	–	–	–	–	–
ANPS_040	Pearson’s *r*	0.239	–	–	–	–	–	–	–
	*p*-Value	<.001	–	–	–	–	–	–	–
ANPS_088	Pearson’s *r*	0.251	0.378	–	–	–	–	–	–
	*p*-Value	<.001	<.001	–	–	–	–	–	–
ANPS_103	Pearson’s *r*	**0.636**	0.266	0.348	–	–	–	–	–
	*p*-Value	**<.001**	<.001	<.001	–	–	–	–	–
ANPS_032	Pearson’s *r*	0.084	−0.095	−0.071	0.067	–	–	–	–
	*p*-Value	.083	.049	.142	.166	–	–	–	–
ANPS_056	Pearson’s *r*	−0.053	−0.271	−0.484	−0.180	0.177	–	–	–
	*p*-Value	.273	<.001	<.001	<.001	<.001	–	–	–
ANPS_072	Pearson’s *r*	−0.134	**−0.709**	−0.314	−0.135	0.135	0.392	–	–
	p-Value	.006	**<.001**	<.001	.005	.005	<.001	–	–
ANPS_111	Pearson’s *r*	−0.215	−0.349	**−0.622**	−0.345	0.098	0.388	0.253	–
	*p*-Value	<.001	<.001	**<.001**	<.001	.044	<.001	<.001	–

**Table 3.** Social dominance item correlations, sample 2, with the correlations of 3 highly correlated pairs of items in bold

		008	040	088	103	032	056	072	111
ANPS_008	Pearson’s *r*	–	–	–	–	–	–	–	–
	*p*-Value	–	–	–	–	–	–	–	–
ANPS_040	Pearson’s *r*	0.234	–	–	–	–	–	–	–
	*p*-Value	<.001	–	–	–	–	–	–	–
ANPS_088	Pearson’s *r*	0.260	0.445	–	–	–	–	–	–
	*p*-Value	<.001	<.001	–	–	–	–	–	–
ANPS_103	Pearson’s *r*	**0.536**	0.261	0.307	–	–	–	–	–
	*p*-Value	**<.001**	<.001	<.001	–	–	–	–	–
ANPS_032	Pearson’s *r*	0.035	0.006	−0.138	−0.137	–	–	–	–
	*p*-Value	.521	.915	.011	.012	–	–	–	–
ANPS_056	Pearson’s *r*	−0.050	−0.333	−0.496	−0.132	0.129	–	–	–
	*p*-Value	.358	<.001	<.001	.015	.017	–	–	–
ANPS_072	Pearson’s *r*	−0.221	**−0.754**	−0.375	−0.191	0.054	0.416	–	–
	*p*-Value	<.001	**<.001**	<.001	<.001	.324	<.001	–	–
ANPS_111	Pearson’s *r*	−0.195	−0.315	**−0.449**	−0.279	0.154	0.292	0.342	–
	*p*-Value	<.001	<.001	**<.001**	<.001	.005	<.001	<.001	–

However, to further statistically investigate these three themes as possible Social Dominance facets, we conducted exploratory factor analyses on both samples. However, the eigenvalue Scree Plot for both samples formed an “elbow” at the second eigenvalue, suggesting that these eight items likely represent a single domain and that the eight Social Dominance items might not segment into a robust and accurate latent factor structure. When we attempted to extract 3 factors using maximum likelihood extraction and oblimin rotation, a Heywood Case (Cooperman & Waller, 2022) defined as a factor loading ≥1 and accounting for 100% or more of the variance appeared in both samples strongly suggesting overextraction. Using principal-axis extraction, a Heywood Case still persisted in sample 1. When two oblique factors were extracted, a communality estimate greater than 1 was encountered during iterations in sample 2, again suggesting overextraction. In sum, we conclude that the themes of liking to be the leader, needing to win, and needing to outperform peers are all sampling from a single Social Dominance domain and may not represent distinct facets.

### 2.3. Statistical analyses

As much of the ANPS data have been already investigated in the Davis and Montag (2024) paper and the data are freely available (see for OSF link: https://osf.io/de7zr/), we focus in the present work on the investigation of the Social Dominance scale and its correlations with the primary emotional systems. We also present associations between Social Dominance and Satisfaction with Life as assessed with Diener’s scale and the Spirituality embedded in the ANPS as these scales could positively or negatively contribute to the expression of Social Dominance. The Social Dominance scale is also tested regarding the variables of age and gender to get deeper insights about associations with relevant person-characteristics. Also, the ANPS 3.1 scales were regressed on the Social Dominance scale to build a statistical model for the expression of Social Dominance. The analyses have been run with the Jamovi package 2.4.8.0 and SPSS 29.0.1.0.

## 3. Results

### 3.1. Age, gender and social dominance

In sample 1 and sample 2 interestingly no gender differences in Social Dominance could be observed (in both samples slightly failing significance: *t*_(423)_ = 1.89, *p* = .059 and *t*_(336)_ = 1.74, *p* = .083 with small effect sizes in both samples: 0.19). See also Table 4 for illustrations. In both samples higher age went along with lower Social Dominance: sample 1: *r* = −.22, *p* < .001 and sample 2: *r* = −.18, *p* < 001.

**Table 4.** Descriptive statistics for the social dominance scale

Social dominance	Total sample	Males	Females	*T*-Tests
Sample 1	*M* = 3.25 (SD = 0.80)	*M* = 3.34 (SD = 0.79)	*M* = 3.19 (SD = 0.79)	*t*_(423)_ = 1.89, *p* = .059, Cohen’s *d* = 0.19
Sample 2	*M* = 3.47 (SD = 0.82)	*M* = 3.56 (SD = 0.80)	*M* = 3.41 (SD = 0.82)	*t*_(336)_=1.74, *p* = .083, Cohen’s *d* = 0.19

### 3.2. Primary emotional systems and social dominance in sample 1 and sample 2

As can be seen in the correlation patterns shown in Table 5 most dimensions show at best weak associations with Social Dominance. The most robust association can be observed between ANGER and Social Dominance in both samples (sample 1 = 0.36, *p* < .001 vs. sample 2 = 0.42, *p* < .001). To a lesser extent higher CARE went along with lower Social Dominance tendencies (sample 1 = −0.15, *p* = .001 vs. sample 2 = −0.22, *p* < .001). Correlations between SEEKING and Social Dominance also reached statistical significance in both samples.

**Table 5.** Pearson correlations between social dominance, primary emotional systems, spirituality and satisfaction with life

	Association with social dominance (sample 1)	Association with social dominance (sample 2)
SEEKING	*r* = .13, *p* = .007	*r* = .12, *p* = .035
FEAR	*r* = .09, *p* = .06	*r* = .14, *p* = .011
CARE	*r* = −.15, *p* = .001	*r* = −.22, *p* < .001
ANGER	*r* = .36, *p* < .001	*r* = .42, *p* < .001
PLAY	*r* = .08, *p* = .084	*r* = .01, *p* = .808
SADNESS	*r* = .05, *p* = .35	*r* = .07, *p* = .202
Spirituality	*r* = −.12, *p* = .018	*r* = −.10, *p* = .057
Satisfaction with Life	*r* = −.04, *p* = .404	*r* = −.083, *p* = .129

*p*-Values reported on two-tailed level.

### 3.3. Predicting social dominance via regression analysis

Hierarchical regression analyses may offer a suggestion on how to better interpret these correlations. Two such regression models were computed (one for sample 1 and one for sample 2) with Social Dominance being the outcome variable. In block 1 age and gender were included as sociodemographic variables and in block 2 all primary emotional systems. Both in sample 1 (*F*_(8,416)_ = 15.3, *p* < .001, 22,7% explained variance) and sample 2 (F_(8,329)_ = 13.1, *p* < .001, 24,1% explained variance) we found support for the role of high ANGER, high SEEKING and low CARE for explaining Social Dominance. See Tables 6 and 7.

**Table 6.** Model coefficients: predicting social dominance in sample 1 via a hierarchical regression model (significant primary emotional systems marked in bold color)

Predictor	Estimate	SE	***T***	***p***
Intercept^a^	14.5668	3.0975	4.703	<.001
Age	−0.0653	0.0187	−3.501	<.001
Gender:				
2–1	−1.1809	0.5958	−1.982	0.048
**SEEKING**	**2.0952**	**0.5037**	**4.160**	**<.001**
FEAR	0.1154	0.4049	0.285	0.776
**CARE**	**−1.5246**	**0.4798**	**−3.177**	**0.002**
**ANGER**	**2.6669**	**0.3767**	**7.079**	**<.001**
PLAY	0.5015	0.4490	1.117	0.265
SADNESS	0.0923	0.4966	0.186	0.853

a Represents reference level; gender (2: female, 1: male).

**Table 7.** Model coefficients: predicting social dominance in sample 2 via a hierarchical regression model (significant primary emotional systems marked in bold color)

Predictor	Estimate	SE	***T***	***p***
Intercept^a^	19.8245	3.6350	5.454	<.001
Age	−0.0855	0.0267	−3.197	.002
Gender:				
2–1	−0.4043	0.6684	−0.605	.546
**SEEKING**	**1.5888**	**0.5385**	**2.950**	**.003**
FEAR	0.2252	0.4553	0.495	.621
**CARE**	**−1.5955**	**0.5319**	**−2.999**	**.003**
**ANGER**	**2.5673**	**0.3788**	**6.778**	**<.001**
PLAY	0.1193	0.4783	0.249	.803
SADNESS	0.1143	0.5461	0.209	.834

a Represents reference level; gender (2: female, 1: male).

## 4. Discussion

The present study aimed to understand how Social Dominance would be linked to Panksepp’s primary emotional systems. In both investigated samples we observed that higher Social Dominance went along with higher ANGER (moderate effect size), higher SEEKING (low effect size) and lower CARE (low effect size). These observations were confirmed by regression models.

### 4.1. ANGER, SEEKING, CARE and social dominance

The most robust correlation in the present work was the link between ANGER and Social Dominance. However, that these two biopsychological domains are not identical comes from evidence to the contrary showing that primary ANGER is an affectively aversive emotion that animals will turn off given the opportunity (Panksepp, 1998). By contrast, both non-human mammals and humans willingly engage in exerting Social Dominance control over conspecifics. Furthermore, testosterone, which can contribute to Social Dominance is also associated with positive affect (Van Honk et al., 2010). In addition, some brain manipulations such as lateral septal lesions increase RAGE but decrease intermale aggression (Wong et al., 2016) suggesting that dominance and inter-male aggression are distinct from ANGER. We hypothesize that in the development of Social Dominance the expression of ANGER in the context of Social Dominance becomes a *learned instrumental means* for acquiring and retaining control of sought after objects or opportunities (SEEKING), in effect becoming a *learned urge for Social Dominance*. To further support this idea, we briefly highlight the developmental expression and phylogenetic progression of Social Dominance in rat pups, dog puppies, macaques, chimpanzees and humans.

### 4.2. Expressions of social dominance in the mammalian world

One aspect of Social Dominance that is often not considered in a biologically-oriented discussion of Social Dominance is that Social Dominance usually emerges early in development, long before adolescence and the associated increases in testosterone and testosterone/cortisol ratios (van der Westhuizen & Solms, 2015) come into play. Indeed, Social Dominance emerges in the “toddlers” of many species from rats to dogs, monkeys, higher primates, and humans. For example, Panksepp has written that young rat pups that transition into needing to “win” play bouts all the time, namely dominate other rat pups, become undesirable play partners (Panksepp & Biven, 2012, p. 359). Even rat pups do not like to interact with bullies.

In his classic work at The Jackson Laboratory on the genetics and social behavior of dogs, J. P. Scott developed a bone dominance test. Starting at five weeks of age puppies began to control a fresh bone and to aggressively assert control over the bone. The collective data from the 13-year program showed there were large increases of complete dominance control in puppies at 11 weeks in all breeds tested except for wire-haired terrier puppies that continued increasing complete dominance control over a bone until 15 weeks of age – long before sexual maturity at about 7 or 8 months (Scott & Fuller 1965). The dissociation of dominance from testosterone is evidence that Social Dominance is more likely an “instrumentally” learned emergent behavior than a genetically built-in primary emotion.

### 4.3. Macaque monkeys and chimpanzees

In our primate cousins, Social Dominance involves more learning than the standard hierarchical dominance model. Remarkably, dominance rank in rhesus monkeys is basically “socially inherited” from the mother (Suarez-Jimenez et al., 2013). In laboratory-reared rhesus macaques, maternal rearing for eight months was enough time for infant rhesus monkeys to “learn” their relative ranks, and social rank will typically remain stable when separated from the mother (Wooddell et al., 2017). However, free-ranging male rhesus monkeys leave their troop around 5 years of age when they reach sexual maturity and must attempt to transfer their learning to a new troop. Higham and Maestripieri (2010) reported an example of how learned social skills might work in a study of free ranging rhesus macaques in which middle-ranking males formed “revolutionary” social coalitions, which resulted in changes in their dominance hierarchy including the ouster of an alpha male. A study of Assamese macaques confirmed that strong social bonds were linked to coalition formation, which in turn predicted future Social Dominance and influenced paternity success (Schülke et al., 2010). In the same paper, Schulke’s group further reported a study of Barbary macaques showing that social bonding between males in the non-mating season predicted agonistic coalition formation against other males during the mating season. The combination of these studies suggest that the capacity of macaque males to form strong social relationships – perhaps based on social learning during their pre-sexual maturity years with their mother – is material to their Social Dominance ranking among males as well as their paternity success.

The theme that Social Dominance is largely a learned secondary emotional process facilitated by social bonding continues with chimpanzees. But with chimpanzees, most females (not males) leave their natal group when they reach puberty and join another community. And, as Reddy and colleagues point out “As adolescent female chimpanzees are attempting to integrate [into a new community] they are often navigating situations that are akin to making first-impressions – where the stakes appear to be extremely high.” (Reddy et al., 2022, p. 17). Data collected over a 35 year period in Gombe National Park on 14 females that had been observed for at least 12 years showed that (1) dominance rank was not related to weight, (2) dominance rank increased with age, (3) the female’s rank at age 21 strongly predicted her rank 10 years later, suggesting that early dominance rank advances were important. Further, the dominance rank of mothers was significantly correlated with their infants surviving to age 5 demonstrating the fitness advantages high ranking chimpanzee mothers provide their offspring (Pusey et al., 1997).

Chimpanzee males do not disperse into new communities and are further able to reap the social advantages of being reared by high-ranking mothers. This can include being exposed to high-ranking males that adolescents know well because these males shared close bonds with the adolescents’ mothers and who may be fathers or act as father figures (Sandel et al., 2020). Such exposure to high ranking males offers important social connections as well as social learning opportunities.

Exploring male social relationship more fully, Gilby et al. (2013) analyzed 14 years of genetic and behavioral data from Jane Goodall’s group in Gombe National Park, Tanzania (divided into two-year periods). They found significant connections between coalitions, social network connections, and increasing Social Dominance rank as well as the probability of siring at least one offspring within the same two-year period. Emphasizing the importance of social relationships on reproductive fitness, while males with the highest rate of “solo aggression” towards other males were the most likely to increase in social rank, the rate of solo aggression had no effect on the number of sired offspring (Gilby et al., 2013) suggesting that “social relationship skills” may be a better fitness indicator in male chimpanzees than raw “agonistic skills.” Accentuating the importance of the close, prosocial, cooperative nature of such male social skills, Feldblum et al. (2021) used genetic data to confirm previous findings (Bray, Pusey & Gilby, 2016; Duffy, Wrangham & Silk, 2007) that alpha male chimpanzees granted “mating concessions” to subordinate males that frequently “groomed” them and “offered coalition support” to the alpha male.

Overall, it is increasingly clear that learning social skills and forming close social bonds underlie the formation of chimpanzee social hierarchical organization both for dispersing females and males maturing in their natal social community. Especially for chimpanzee males, maximizing the acquisition of important life resources (high SEEKING) in a self-referential manner (low CARE) requires expressing agonistic behavior (high ANGER) using learned social skills in the context of prosocial cooperative relationships. In other words, with primate increased social complexity supported by increased cortical capacity (Dunbar & Shultz, 2007), it is more learned social competency and less raw coercive force that contributes to effective Social Dominance and reproductive fitness.

Hence, the Social Dominance equation becomes how to optimize the acquisition of important life resources (SEEKING). The answer for alpha chimpanzee male reproductive success seems to be “self-handicapping” your own mating capacity and grant mating concessions to other high ranking subordinates in exchange for the coalition support required to maintain and even extend alpha status.

### 4.4. Human children

Consistent with other mammals, human preschool children begin learning dominance hierarchies early in life and form hierarchies over access to toys, for example (Thomsen, 2020). One illustration studied pairs of five-year old children in a game of “chicken.” These pairs of preschoolers coordinated their encounters such that the child who received the highest reward was the child who had previously held a toy the longest, and who was also rated by their teacher as the more dominant (Grueneisen & Tomasello, 2017).

However, older children generally begin to shift dominance strategies as they mature into less coercive and more socially acceptable approaches (Teisl et al. 2012). Teisl et al. (2012) designed a one-week summer day camp project to measure family influences on the shift from coercive to more prosocial influence strategies. Specifically, 220 children including 72 physically and/or sexually abused children and 148 physically neglected/emotionally maltreated children balanced by 220 demographically comparable but non-maltreated children between 6 and 13 years of age were invited to the day camp.

However, the Teisl et al. (2012) paper takes the dominance discussion a step further by linking coercive aggression to early childhood emotional maltreatment and neglect. It was the emotionally maltreated and neglected children that were more likely to be rated coercive rather than socially competent. Yet, it wasn’t that maltreated children were rated as less dominant than the non-maltreated group. It was just that their dominance was expressed differently. Camp counselor’s classifications of children as coercively dominant were rated by their camp peers as disruptive and aggressive. On the other hand children rated by counselors as competently dominant were rated by their camp mates as leaders and cooperative. Correlationaly, they linked coercively dominant behavior to childhood maltreatment and neglect. Further, it is tempting to hypothesize that cases of coercive dominance observed in chimpanzees could also be linked to early neglect or abuse.

To summarize this discussion, the cross-species review of Social Dominance suggested that the exercise of Social Dominance in mammals such as rats and dogs emerged as coercive dominance. However, a more complex model requiring social skills for developing new cooperative relationships and coalitions emerged in primates including humans as a prosocial strategy for increasing access to important life resources.

We provided further evidence that a core “dominance” construct focused on acquiring and retaining life resources is learned by all young mammals, but with larger brained primates such as chimpanzees, and humans, one must additionally specify how dominance will be expressed: coercively, through anger and intimidation – force or the threat of force – or prosocially through social bonding and relationship competency – partnership and cooperation. Particularly in larger brained species, the extent to which these styles of gaining desired resources are learned through early rearing experiences remains a provocative and socially important question that is not yet well specified.

### 4.5. Limitations

The present study comes with the usual limitations, such as relying on self-report and not including biological correlates. Further, the present study is of cross-sectional nature, hence no causality can be inferred based on the present data. However, we have provided some evidence that the expression of Social Dominance in mammals could be more an instrumentally learned behavior that becomes increasing sophisticated in large-brained mammals such as chimpanzees and humans. Further research on the impact of early rearing on the coercive or prosocial expression of Social Dominance would contribute to the understanding of child development as well as the treatment of excessive social aggression. Also, replications of our data findings would further illuminate the role of the ANGER system in Social Dominance. One related hypothesis might be that the prosocial styles of social influence might be facilitated by sensitizing and strengthening the CARE system to better counterbalance ANGER with CARE. Specifically, more research into the relative effectiveness of organizational leadership styles reflecting more relationship building (effective listening and regulating anger expression when frustrated – more caring) versus more coercive (openly directive and assertive – more angry) would test the hypothesis that how leaders express their Social Dominance matters.

The study also relies on convenience samples, therefore revisiting the present associations in further representative, but also clinical samples might result in further important insights. This study is further limited by the inability to statistically identify clear social dominance facets, which needs to be revisited again in the future. Finally, another limitation of this study is that the current ANPS 3.1 Social Dominance scale does not differentiate between the coercive and prosocial dominance styles. Determining such a distinction remains for future research.

## 5. Conclusions

We replicated robust associations between ANGER and Social Dominance (positive), SEEKING and Social Dominance (positive) and CARE and Social Dominance (negative) in two samples. This finding is consistent with a learning model of Social Dominance. The learning model is reinforced by chimpanzees learning to rely on close cooperative social relationships rather than raw agonistic capacities to optimize the acquisition of life resources. Likewise, humans as they mature, can also increasingly rely on social cooperation rather than coercive means to reach their goals. These findings provide new insights into the nature of expressed social dominance as measured by the ANPS Social Dominance scale.

In short, we support the hypothesis that large-brained species optimize the acquisition of important life resources (high SEEKING) self-referentially (low CARE) by expressing agonistic behavior (high ANGER) using learned social skills in the context of prosocial cooperative relationships that increasingly incorporate the needs of others.

## Supporting information

10.1017/pen.2026.10007.sm001 Davis and Montag supplementary material Davis and Montag supplementary material

## Data Availability

The data underlying this work can be found at the Open Science Framework: https://osf.io/de7zr/

## Supplementary material

The supplementary material for this article can be found at https://doi.org/10.1017/pen.2026.10007
