# Supplementary material for: Shedding light on social dominance within the affective neuroscience personality scales
Source: Personal Neurosci. 2026 Jun 2;9:e3. doi: 10.1017/pen.2026.10007 (PMC13244340; doi:10.1017/pen.2026.10007)
Supplement: Davis and Montag supplementary material [file S2513988626100078sup001.docx]

Supplementary Material

| STable 1: Correlations of Social Dominance without item 32 and primary emotional systems in sample 1 | | | |
| --- | --- | --- | --- |
|  |  | Social Dominance |  |
| SEEKING | Pearson's r | 0.160 |  |
|  | p-value | <.001 |  |
| FEAR | Pearson's r | 0.072 |  |
|  | p-value | .140 |  |
| CARE | Pearson's r | -0.133 |  |
|  | p-value | .006 |  |
| ANGER | Pearson's r | 0.332 |  |
|  | p-value | <.001 |  |
| PLAY | Pearson's r | 0.094 |  |
|  | p-value | .053 |  |
| SADNESS | Pearson's r | 0.032 |  |
|  | p-value | .508 |  |

| STable 2: Correlations of Social Dominance without item 32 and primary emotional systems in sample 2 | | | |
| --- | --- | --- | --- |
|  |  | Social Dominance |  |
| SEEKING | Pearson's r | 0.113 |  |
|  | p-value | .038 |  |
| FEAR | Pearson's r | 0.150 |  |
|  | p-value | .006 |  |
| CARE | Pearson's r | -0.202 |  |
|  | p-value | <.001 |  |
| ANGER | Pearson's r | 0.412 |  |
|  | p-value | <.001 |  |
| PLAY | Pearson's r | -0.035 |  |
|  | p-value | .518 |  |
| SADNESS | Pearson's r | 0.090 |  |
|  | p-value | .099 |  |
